# Illinois State Hospital for the Insane

**Published:** 1847-04

**Authors:** 


ARTICLE III.

### ILLINOIS STATE HOSPITAL FOR THE INSANE.

The bill passed at the late session of the legislature incorporating this institution, appoints nine trustees, and empowers them to cause to be erected, upon a farm not to exceed three hundred acres, within four miles of Jacksonville, in Morgan county, suitable buildings and out houses for said institution, and draw upon the treasurer of state from time to time as may be needed in the proscution of said work, for the funds for the insane, which are to be raised by an annual tax of two cents on each one hundred dollars valuation of property in the state. This, we are informed by the secretary of the board, will yield about twenty thousand dollars per annum.

The whole establishment is to provide for the accommodation of two hundred and fifty patients, and the necessary officers, attendants, and servants for its management. The amount of its cost is limited to sixty thousand dollars.

The trustees are to appoint a superintendant for a term of ten years, and fix his salary, which shall not be reduced during his term, who shall be a well educated physician. Section eighth defines his duties as follows: "The superintendent shall appoint and exercise entire control over all subordinate officers and assistants in this institution, and shall have entire direction of the duties of the same."

Pauper patients are to have the preference in admission, who are to be supported by the counties sending them.

The trustees have determined to commence operations in building the ensuing fall, and will, we understand, push the work onward, as humanity most assuredly demands, to a rapid completion.

We hope the trustees will adopt the plan, so generally recommended by those conversant with the subject, of placing a physician, well informed on the subject, in charge of the erection of the building, at least so far as adapting it to the purposes for which it was designed are concerned. It may, and no doubt would, save much expense in making alterations in future, and probably prevent inconveniences that would be irremediable.

